# Impact of Serum Uric Acid Levels on Outcomes following Renal Artery Revascularization in Patients with Renovascular Disease

**DOI:** 10.1155/2019/3872065

**Published:** 2019-01-30

**Authors:** Xiao-jun Chen, Alfonso Eirin, Garvan C. Kane, Sanjay Misra, Stephen C. Textor, Amir Lerman, Lilach O. Lerman

**Affiliations:** ^1^Division of Nephrology and Hypertension, Mayo Clinic, Rochester, MN, USA; ^2^Department of Nephrology, The Second Xiangya Hospital of Central South University, Changsha, Hunan, China; ^3^Department of Cardiovascular Diseases, Mayo Clinic, Rochester, MN, USA; ^4^Division of Vascular and Interventional Radiology, Mayo Clinic, Rochester, MN, USA

## Abstract

**Background:**

Percutaneous transluminal renal angioplasty (PTRA) improves blood pressure (BP) and renal function only in selected patients with atherosclerotic renovascular disease (ARVD). Hyperuricemia is associated with elevated risk for hypertension and chronic renal disease, but its role in renovascular hypertension is unclear. We hypothesized that hyperuricemia negatively impacts renal and BP outcomes among patients with ARVD undergoing PTRA.

**Methods:**

This retrospective, observational cohort study included 94 patients with ARVD and preserved systolic cardiac function, who underwent PTRA at Mayo Clinic, Rochester, Minnesota. Renal, BP, and mortality outcomes were compared among patients according to their serum uric acid (SUA) levels. Multivariate analysis was used to determine significant predictors of renal, BP, and mortality outcomes after PTRA.

**Results:**

Compared to patients with normal basal SUA levels (≤5.7 mg/dl), patients with very high SUA (≥8.7 mg/dl) had lower baseline estimated glomerular filtration rate (eGFR), more extensive use of antihypertensive and diuretic drugs, increased baseline systolic blood pressure (SBP), and elevated left ventricular mass index. After PTRA, multiple logistic regression analysis showed that, compared to normal SUA, very high SUA was associated with decreased odds ratio (OR) of change in eGFR (adjusted OR=0.90; 95% confidence interval [CI], 0.86-0.95), but not of change in SBP. In multivariate linear analysis SUA independently predicted delta urine protein/creatinine ratio (*β*: 26.0; 95% confidence interval, 13.9 to 38.1).

**Conclusion:**

Severe hyperuricemia in patients with AVRD may have a negative impact on outcomes of renal revascularization.

## 1. Introduction

Atherosclerotic renovascular disease (ARVD) is a common and progressive disease with manifestation of renovascular hypertension. It affects 7% of individuals older than 65 years and accounts for 90% of cases of renal artery stenosis, resulting in a reduction of renal blood flow (RBF) to the affected kidney [1]. In addition to medical treatment, percutaneous transluminal renal angioplasty (PTRA) became available in the 1990s. However, currently the only class I recommendation for PTRA is hemodynamically significant ARVD and unexplained congestive heart failure or sudden unexplained pulmonary edema [2]. Other than those, results of two large randomized trials [3, 4] demonstrated no significant differences in renal recovery between pharmacological treatment and renal artery stenting. However, since the entry criteria for these two trials were liberal, and smaller single-center trials and observational studies have shown positive clinical outcomes following PTRA [5], specific subgroups might benefit from revascularization [6]. How to identify individuals who will derive clinical benefit from PTRA remains unclear.

Increasing evidence over the past century has supported a strong association between serum uric acid (SUA) and hypertension, independent of traditional risk factors [7]. Notably, the association of SUA with hypertension becomes already evident in the pediatric and adolescent population [8, 9]. Borghi et al. have described that elevated SUA can reduce the blood pressure (BP) response to antihypertensive drugs [10]. Several studies have also revealed a positive association between elevated SUA levels and progression of chronic kidney disease (CKD), independent of estimated glomerular filtration rate (eGFR) [11]. Although the potential efficacy of decreasing SUA in the prevention or control of CKD remains controversial, SUA lowering treatments delay progression of renal disease in patients with CKD in several studies [12, 13]. Therefore, hyperuricemia, hypertension, and CKD progression appear to be linked.

The mechanisms responsible for essential hypertension differ from those contributing to renovascular hypertension, yet the role of SUA in hypertension and renal injury in renovascular disease has not been elucidated. SUA is elevated in patients with ARVD compared to healthy controls [14], but whether hyperuricemia impedes renal function and BP improvement after renal artery revascularization remains unclear. Therefore, this study was designed to test the hypothesis that hyperuricemia negatively impacts renal and BP outcomes among patients with ARVD undergoing PTRA.

## 2. Materials and Methods

### 2.1. Study Population

After receiving approval from the Institutional Review Board of the Mayo Clinic, 94 patients above 18 years of age at Mayo Clinic, Rochester, Minnesota, USA, were enrolled in the present study. Informed written consent was obtained. Patients identified with significant ARVD, using entry criteria analogous to enrolment in CORAL [15], were recruited between January 2004 and August 2012. Patients whose left ventricular ejection fraction (EF) was under 50%, as assessed by cardiac echocardiography performed within a 2-year period, were excluded, given the association of systolic heart failure with SUA [16]. Participants subsequently underwent renal artery revascularization and stenting, following standard clinical protocols. Patients were stratified into the following groups according to SUA level quintiles: normal SUA, ≤ 5.7 mg/dl (20th percentile); moderate-high SUA, 5.7-8.7 mg/dl (20th-80th percentile); and very high SUA, ≥8.7 mg/dl (≥80th percentile). Demographics and outcomes were compared among groups.

### 2.2. Clinical Data Collection and Laboratory Measurements

Baseline clinical parameters were recorded at the time of PTRA. Follow-up was achieved via the electronic medical records within 3 years and with the censored point at the last observed clinical visit at Mayo Clinic, the end of the study period, or death. All echocardiograms were performed by certified technicians following standard clinical procedures and read by level III certified echocardiographers. EF was measured by the quantitative 2-dimensional biplane volumetric Simpson method. Some of the data were only available for some patients, such as level of urine protein/creatinine ratio (PCR) (Table 1). Cardiovascular disease (CAD) was defined as (1) any evidence of coronary atherosclerotic plaque on coronary angiography; (2) ischemia on noninvasive cardiac testing; or (3) history of myocardial infarction, percutaneous coronary intervention, or surgical revascularization. Cerebrovascular disease (CVD) was defined as (1) prior history of ischemic stroke or (2) asymptomatic carotid artery stenosis (>60%). Peripheral arterial disease (PAD) was defined as a history of claudication or an ankle brachial index under 0.90. The eGFR was calculated according to the Modification of Diet in Renal Disease study equation.

### 2.3. Statistical Analysis

Statistical analysis was performed using JMP version 13.0 (SAS Institute, Inc., Cary, NC, USA). Continuous variables are expressed as mean±SD and skewed variables as median (range). Parametric (one-way ANOVA or Student's* t*-test) and nonparametric (Wilcoxon or Kruskal-Wallis) tests were used to compare continuous variables among the groups and paired* t*-tests within groups. Pearson's chi-squared test was used for categorical data. We defined improvement of systolic BP (SBP) as mean reduction by more than ≥5 mmHg after PTRA compared to baseline, which is clinically and statistically meaningful, as done before [17]. Multiple logistic regression analysis was performed to determine the trends of improvement in SBP or change (delta) of eGFR according to groups. Unadjusted and adjusted odds ratios (ORs) were calculated using the normal group as the reference. In the adjusted model, we controlled age, sex, body mass index (BMI), number of antihypertensive drugs, diuretic use, low-density lipoprotein (LDL), baseline eGFR, baseline SBP, and left ventricular mass index (LVMI). Controlled variables were selected based on several criteria. Age, sex, and BMI were first selected after a literature review, and the variables that differed among groups were then contained. Regressions were calculated by the least-squares fit in correlation analysis. Univariate and multivariate analysis with multiple linear or Cox proportional hazard regression model was used to determine significant predictors of urine PCR after PTRA, as well as all-cause mortality. Alpha was set at 0.05, and p value <0.05 was considered to be statistically significant.

## 3. Results

### 3.1. Baseline Patient Characteristics

Table 1 shows the baseline clinical characteristics of 94 patients categorized by baseline SUA into normal SUA, moderate-high SUA, and very high SUA groups. Age, sex, race distribution, BMI, and diastolic BP (DBP) were not different among the groups, which also had similar prevalence of diabetes mellitus, CAD, CVD, and PAD. Patients in both the very high or moderate-high SUA groups were more likely to use a larger number of antihypertensive and diuretic drugs and had elevated baseline SBP compared to the normal SUA group (p<0.05). The severity of renal artery stenosis was similar among the groups, but patients with very high SUA had greater renal damage evidenced by lower eGFR (p<0.01) compared to the other groups, with no difference in urinary PCR among the groups. Patients with very high SUA had lower level of LDL compared to the moderate-high group (p<0.05), but not to the normal group. As per echocardiography, the LVMI was higher in the very high SUA group (p<0.01), whereas systolic and diastolic left ventricular functions were similar among the groups. Mean SUA level was higher in the male (7.7±1.9 mg/dl) than in the female (7.0±1.9 mg/dl) cohorts (p<0.05).

### 3.2. Uric Acid and BP Outcomes

Following revascularization DBP fell similarly in all the groups, whereas SBP only fell in very high SUA group (p<0.05), in which the change in SBP was greater than in the normal group (Table 1, p<0.05). The association between severe hyperuricemia and improvement of SBP was statistically significant in the unadjusted model (OR 2.19 [95% confidence interval {CI} = 1.52-3.16, p=0.03]), but lost significance after adjustment (Table 2).

### 3.3. Uric Acid and Renal Outcomes

Significant falls in eGFR were observed only in very high SUA group (Table 1, p<0.01), which therefore remained lower with a greater gap compared to other groups. The association between SUA and loss of renal function was significant both before and after adjustment. Patients in very high SUA group had a lower OR (Table 2, 0.9 [95% CI=0.86-0.95, p<0.05]) for change (delta) of eGFR after adjusting for age, sex, BMI, number of antihypertensive drugs, diuretic use, LDL, baseline eGFR, baseline SBP, and LVMI.

### 3.4. Predictors of Proteinuria after PTRA

Data available in 69 patients showed that the delta change in urine PCR after PTRA directly correlated with baseline SUA (R=0.3, p<0.05). Patients using *α*-blocker had a greater change in urine PCR compared to nonusers (p<0.01). A multivariate linear analysis revealed that SUA independently predicted delta PCR (*β*: 26.0; 95%CI: 13.9 to 38.1,), as did use of *α*-blockers (*β*:-98.9; 95%CI: −136.8 to -61.0) (Table 3).

### 3.5. Follow-Up Survival Data

Timing of the last follow-up after revascularization was similar among the groups (Table 1). A univariate Cox analysis showed that age, sex, baseline SUA, lower LDL, and eGFR were significantly associated with mortality (Table 4). A multivariate Cox analysis revealed that only age (HR per 1 year: 1.08; 95% CI, 1.02-1.14) and pre-PTRA eGFR (HR per 1 year: 0.97; 95% CI, 0.94-1.00) were independent predictors of mortality (Table 4).

## 4. Discussion

We evaluated the associations of serum uric acid levels with outcomes of renal revascularization in AVRD patients. Our study shows that renal function in patients with severe hyperuricemia may benefit less from renal revascularization than those with normal SUA, given that very high SUA was associated with a decreased odds ratio of a rise in eGFR. Our findings in renovascular disease are consistent with the role of hyperuricemia as a risk factor for incident or progression of CKD [18]. On the other hand, patients with very high SUA showed a greater fall in SBP, although a relationship between hyperuricemia and BP outcome was not observed after adjustments in our subjects.

Renovascular hypertensive patients with severe hyperuricemia had higher BP and left ventricular hypertrophy, indicated by increased LVMI, which may result from activation of the renin-angiotensin system [19], reduction of vascular nitric oxide production, or activation of distal nephron sodium channels [20]. Of note, even patients with moderate to high levels of SUA were likely to use a larger number of antihypertensive and diuretic drugs compared to patients with normal SUA. These observations are congruent with former observational studies, which suggested that hyperuricemia is associated independently with both hypertension and diuretic use [21]. Furthermore, a prospective study showed that diuretic use raises risk for gout in hypertensive patients with no histories of gout at baseline [22]. Hence, clinicians should be also cautious when choosing first-line antihypertensive drugs to treat ARVD-associated hypertension to consider this potential side effect of diuretic drugs. Interestingly, a fall in SBP after PTRA was only observed in patients with severe hyperuricemia, contrasting our hypothesis. This may have been related to the basal SBP, given that the relationship between severe hyperuricemia and improvement in SBP became insignificant after correction of baseline SBP. Unavailability of the number of antihypertensive drugs taken in each group at follow-up, an index for BP outcomes, limits interpretation of the role of SUA levels on the improvement in BP. Moreover, while the groups had a similar degree of stenosis based on CT/MRI and Doppler, Renal Resistive Index was unavailable to establish the hemodynamic significance of ARVD.

In several studies, renal function in patients with ARVD that was deteriorating before PTRA stabilized thereafter. Ramos et al. [23] studied 105 patients with ARVD over one year following PTRA and found a significant increase in GFR (from 33.3±10 to 54±24 ml/min/1.73m^2^) in a subgroup of patients with an initially lower eGFR. In another prospective, single-arm, multicenter clinical study [24], and PTRA stabilized renal function in 108 ARVD patients at 12 months (from 40.7±10 to 40.8±13 ml/min/1.73m^2^). Conversely, in the present study we observed a significant fall in eGFR in patients with very high SUA group, whose baseline eGFR (41.2±15.8 ml/min/1.73m^2^) was very close to the previous studies. Severe hyperuricemia was associated with greater renal dysfunction following renal artery revascularization even after adjustment for baseline renal function. These findings imply that UA may blunt renal recovery in renovascular hypertensive patients independent of baseline renal function. Renal function in normal SUA group was unchanged, although we cannot rule out that PTRA improves GFR in stenotic and decreases it in contralateral, nonstenotic kidneys [25].

Furthermore, SUA remained an independent predictor for increased proteinuria after adjustment. Proteinuria not only predicts worse renal outcome but is also associated with an increased risk for cardiovascular disease [26]. Several studies have demonstrated an independent association between hyperuricemia and proteinuria in the general population [27]. Elevated UA levels could increase generation of reactive oxygen species concomitant with UA formation by xanthine oxidase [28], which increase oxidative stress in glomeruli and result in endothelial dysfunction and podocytes injury [29, 30], leading to proteinuria. Consistent with this speculation, lowering UA treatment with xanthine oxidase inhibitors reduces proteinuria in patients with CKD-3 [31]. Furthermore, *α*-blocker usage was associated with decreased PCR after PTRA, consistent with previous study in which doxazosin reduced proteinuria by 34% [32] in patients with hypertension.

We also found that baseline SUA was associated with, but did not independently predict, all-cause mortality after PTRA. A relationship between UA and mortality has been previously shown in stages 3-5 CKD patients [33], but our study included a smaller number of patients with relatively mild CKD and a smaller range of SUA levels and excluded patients with overt heart failure. Hence, the cause-effect relationship between SUA and changes in GFR in patients with ARVD needs to be pursued in larger studies,

This study has some limitations. First, this small single-center retrospective study included primarily Caucasian patients, limiting the generalizability of the results. Second, although we observed no significant difference in follow-up time from revascularization to the outcomes studied among groups, the pertinent conclusion would have been more robust had the outcomes been studied with a scheduled timetable. Furthermore, we excluded patients with severe cardiac dysfunction, which may introduce selection bias, because UA is associated with increasing incident heart failure in elderly people [16] and severe heart failure per se may worsen renal failure and hypertension. However, this exclusion might have also limited the range of SUA and morbidity in our patient cohort. Lastly, SUA may vary due to dietary and medications individually; therefore, a single basal SUA measurement may underestimate its importance in BP and renal outcomes after PTRA. The reason for the association of lower LDL levels with SUA in the upper quintile group is unclear but may potentially involve greater use of xanthine oxidase inhibitor in this group [34], the data of which is unavailable in our study.

## 5. Conclusion

This study shows that severe hyperuricemia may be associated with greater residual renal dysfunction and increased proteinuria after PTRA in ARVD patients, whereas no significant relationship between hyperuricemia and BP outcome was observed. Thus, severe hyperuricemia in AVRD patients may have a negative impact on the outcomes of renal revascularization, although this preliminary finding requires confirmation in larger clinical trials. Further studies are needed to explore the use of severe hyperuricemia as an exclusion criterion for PTRA, or the utility of a UA lowering drug before intervention.

## Figures and Tables

**Table 1:** Clinical characteristics of 94 patients with ARVD stratified by SUA level before and after PTRA.

**Variables**	**Normal**	**Moderate-high**	**Very High**
**N, number (**%**)**	14 (15)	58 (62)	22 (23)
**PTRA follow-up (years)** ^**a**^	0.22 (0.003-1.8)	0.22 (0.005-2.6)	0.44 (0.008-2.9)
**Survival follow-up (years)** ^**b**^	4.5 (0.45-8.4)	7.2 (0.70-8.5)	4.0 (0.72-7.3)
**Age (years)**	78 (58-91)	75 (51-87)	75 (67-91)
**Sex female/male**	10/4	31/27	11/11
**BMI (kg/m** ^**2**^ **)**	28 (20-40)	28 (21-48)	32 (23-39)
**Race (white/other/unknown)**	13/1/0	55/0/2	21/0/1
**Uric acid, mg/dl**	5 (4-5)	7 (6-8)	10 (9-13)
**Total cholesterol (mg/dl)**	192 (109-223)	169.5 (93-382)	149 (88-329)
**LDL (mg/dl)**	96.5 (42-133)	93 (45-225)	78 (42-198) ^†^
**HDL (mg/dl)**	51.5 (24-103)	49 (26-83)	45 (28-80)
**Triglycerides (mg/dl)**	126.5 (55-222)	143.5 (52-494)	150.5 (48-335)

**Comorbidities**			
**Diabetes mellitus, n (**%**)**	2 (14)	14 (24)	7 (32)
**Coronary artery disease, n (**%**)**	5 (36)	34 (59)	14 (63)
**Cerebrovascular disease, n (**%**)**	6 (43)	30 (52)	12 (55)
**Peripheral artery disease, n (**%**)**	6 (43)	21 (36)	5 (23)
**Recent myocardial infarction/stroke, n (**%**)**	0 (0)	4 (7)	3 (14)
**Hospitalization for pulmonary edema, n (**%**)**	2 (14)	6 (10)	2 (9)
**Coronary artery bypass grafting, n (**%**)**	3 (21)	23 (72)	6 (27)
**Atrial fibrillation, n (**%**)**	4 (29)	17 (29)	7 (32)
**Sleep apnea, n (**%**)**	5 (36)	16 (28)	8 (36)

**Smoking status**			
**Current smoker, n (**%**)**	1 (7)	3 (5%)	2 (9%)
**Nonsmoker, n (**%**)**	13 (93)	55 (95%)	20 (90%)

**Arterial hypertension (mmHg):**			
**SBP at baseline**	138 ± 22	149 ± 24*∗*	153 ± 17*∗*
**SBP at follow-up**	140 ± 16	143 ± 18	142 ± 15^‡^
Δ**change of SBP**	-1 ± 18	-5 ± 25	-11 ± 16*∗*
**DBP at baseline**	72 ± 10	75 ± 14	73 ± 12
**DBP at follow-up**	65 ± 9^‡^	68 ± 12^‡^	62 ± 12^‡^
Δ**change of DBP**	-7 ± 8	-6 ± 15	-10 ± 13
**Antihypertensive drugs at baseline (number)**	2 (1-5)	4 (1-7)*∗*	3.5 (2-6)*∗*
**ACE inhibitor or ARB at baseline, n (**%**)**	9 (64)	43 (74)	16 (73)
**Diuretics, n (**%**)**	7 (50)	46 (79)*∗*	19 (86)*∗*
**β-blocker, n (**%**)**	10 (71)	49 (84)	15 (68)
**α-blocker, n (**%**)**	1 (7)	5 (9)	4 (18)
**Calcium channel blocker, n (**%**)**	6 (43)	26 (45)	12 (55)
**Statins**	17 (77)	43 (74)	10 (71)

**Renal function **			
**Baseline eGFR, ml/min/1.73m** ^**2**^	52.9 ± 10.9	48.0 ± 15.9	41.2 ± 15.8*∗*^†^
**Follow-up eGFR, ml/min/1.73m** ^**2**^	51.9 ± 14.4	47.2 ± 17.8	35.0 ± 15.7*∗*^†‡^
Δ**change of eGFR, ml/min/1.73m**^**2**^	1.2 ± 15.7	-0.8 ± 10.9	-6.3 ± 11.4*∗*^†^
**Proteinuria/creatinine** ^**§**^			
**Baseline**	1150 (327-11692)	1081 (320-7043)	1606 (320-7044)
**Follow-up**	861 (455-8219)	1018 (191-7600)	1211 (207-13239)
Δ**change**	-245 (-3473-+2477)	-33 (-7354-+4492)	-180 (-4562-+9215)
**Bilateral RAS, n (**%**)**	3 (27)	13 (25)	6 (29)
**Ultrasound-Doppler peak systolic velocity**	319 ± 32	320 ± 24	315 ± 29
**Grading of stenosis as per CT/MRA** ^||^			
**Moderate, n (**%**)**	4 (31.8)	12 (23)	3 (14.3)
**High grade, n (**%**)**	8 (61.5)	33 (63.5)	15 (71.4)
**Severe, n (**%**)**	1 (7.7)	7 (13.5)	3 (14.3)

**Echocardiographic parameters**			
**Ejection fraction (**%**)**	67 ± 8	65 ± 6	63 ± 6
**Cardiac index (l/min/m** ^**2**^ **)**	2.8 (1.9-4.6)	2.8 (2.0-4.6)	3.0 (2.3-4.5)
**E/E' ratio** ^||^	11.4 (6.7-25)	15 (1-46.7)	17.5 (10-27.5)
**LV mass index (g/m** ^**2**^ **)**	86.5 (74-123)	103 (57-190)*∗*	113 (57-237)*∗*^†^

ARVD, atherosclerotic renovascular disease; PTRA, percutaneous transluminal renal angioplasty; SUA, serum uric acid; BMI, body mass index; SBP, systolic blood pressure; DBP, diastolic blood pressure; HDL, high-density lipoprotein; LDL, low-density lipoprotein; CAD, coronary artery disease; ACEI, angiotensin-converting-enzyme inhibitor; RAS, renal artery stenosis; CT, computed tomography; MRA, magnetic resonance angiogram; E, peak mitral inflow velocity; E', medial mitral annulus peak diastolic velocity; eGFR, estimated glomerular filtration rate; LV, left ventricular.

^a^ Follow-up duration for revascularization studies. ^b^ Follow-up duration for survival analysis.

Data are presented as median (range), N (%), or mean ± SD, as appropriate.

*∗*p<0.05 versus patients with normal SUA. †p<0.05 versus patients with moderate-high SUA. ‡ p<0.05 versus baseline.

^§^n=10 patients with normal SUA, 42 with moderate-high SUA, and 17 with very high SUA. ^||^n=13 patients with normal SUA, 52 with moderate-high SUA, and21 with very high SUA.

**Table 2:** The association between serum uric acid level and improvement in SBP and Δchange in eGFR post-revascularization.

Group	Odds ratio (95% confidence interval) for improvement in SBP			
	Unadjusted	P value	Adjusted*∗*	P value
**Normal **	1.00	NA	1.00	NA
**Moderate-high**	1.54 (1.13-2.10)	0.30	2.04 (1.14-3.66)	0.13
**Very High**	2.19 (1.52-3.16)	0.03	2.61 (1.34-5.09)	0.11
	Odds ratio (95% confidence interval) for Δchange in eGFR, per SD			
	Unadjusted	P value	Adjusted*∗*	P value
**Normal **	1.00	NA	1.00	NA
**Moderate-high**	0.98 (0.96-1.00)	0.37	0.97 (0.95-1.00)	0.19
**Very High**	0.94 (0.91-0.96)	0.029	0.90 (0.86-0.95)	0.03

*∗* Adjusted for age, gender, BMI, number of antihypertensive drugs, diuretic use, LDL, baseline estimated glomerular filtration rate (eGFR), baseline systolic blood pressure (SBP), and left ventricular mass index (log-transformed)

**Table 3:** Multiple linear regression analysis for predictors of delta urine protein/creatinine ratio pre- and post-percutaneous transluminal renal angioplasty (n=69).

	Coefficient estimate	Standard Error	p value
**Uric acid**	26.0	12.1	0.04
**Use of ** **α** **-blocker **	-98.9	37.9	0.02
**Age **	-0.8	2.6	0.77
**Gender female**	7.6	25.5	0.76
**Follow-up years**	24.1	40.9	0.56

**Table 4:** Predictors of Mortality in Patients with ARVD after PTRA.

	Univariate predictors of mortality *∗*		Multivariate cox regression model †	
	HR (95%CI)	p value	HR (95%CI)	p value
**Age, per 1-year increase**	1.08 (1.03-1.14)	0.0007	1.08(1.02-1.14)	0.007
**Male sex**	0.84(0.69-1.00)	0.046	1.00(0.40-2.46)	1.00
**Pre-PTRA eGFR, per SD**	0.96(0.93-0.99)	0.0048	0.97(0.94-1.00)	0.023
**LDL, per SD**	0.99(0.97-1.00)	0.032	0.99(0.97-1.00)	0.062
**Uric acid, per SD**	1.25(1.04-1.49)	0.020	1.10(0.90-1.33)	0.36

PTRA, Percutaneous Transluminal Renal Angioplasty; BP, blood Pressure; SBP, systolic BP; DBP, diastolic BP; eGFR, estimated glomerular filtration rate. LDL, low-density lipoprotein.

*∗* Based on univariate cox regression model. † Based on multivariate cox regression model adjusted for all clinical variables with hazard ratio and 95% confidence interval.

## Data Availability

The data used to support the findings of this study are available from the corresponding author upon request.
